# From gene-for-gene to NLRome-for-effectorome: decoding the dynamic interplay between phytopathogenic effector networks and plant immune systems

**DOI:** 10.1093/femsre/fuag019

**Published:** 2026-05-20

**Authors:** Bingbing Xue, David S Guttman, Lifang Ruan

**Affiliations:** State Key Laboratory of Agricultural Microbiology, College of Life Science and Technology, Huazhong Agricultural University, Wuhan 430070, China; Department of Cell & Systems Biology, University of Toronto, Toronto, Ontario M5S 3B2, Canada; Centre for the Analysis of Genome Evolution & Function, University of Toronto, Toronto, Ontario M5S 3B2, Canada; State Key Laboratory of Agricultural Microbiology, College of Life Science and Technology, Huazhong Agricultural University, Wuhan 430070, China

**Keywords:** plant pathogens, type III effectors, effector-triggered immunity (ETI), nucleotide-binding, leucine-rich repeat immune receptors (NLRs), co-evolution, effectorome, NLRome

## Abstract

Phytopathogenic bacteria rely on type III effectors to suppress host immunity and facilitate colonization. While necessary for virulence, effectors can also trigger effector-triggered immunity (ETI) if hosts have appropriate NLR immune receptors, resulting in a coevolutionary arms races driving high diversity in both the pathogen “effectorome” and the host “NLRome.” Here, we synthesize current knowledge of effectoromes into an “interconnected module model”, that emphasizes how functional redundancy among effectors organizes them into modules targeting shared host processes; how low target specificity creates interconnections between these modules; how effector–effector interactions can modify infection outcomes; and how the cumulative presence of multiple ETI-eliciting effectors within a repertoire generates an overall ETI load that constrains pathogen fitness. This systems-level perspective reframes the classical gene-for-gene model into a dynamic NLRome–effectorome model characterized by quantitative ETI responses whose magnitude and ultimate outcome is a dynamic balance between ETI load and suppression. Advancing disease resistance requires strategies that exploit this equilibrium, including rational stacking of NLRs targeting core, conserved effectors. Such approaches highlight the potential of network-based frameworks for designing durable, broad-spectrum crop immunity.

## Teaser

We synthesize recent developments in effector-mediated host-microbe interactions into an interconnected, systems-level view of phytobacterial effectoromes, in which functional redundancy, low target specificity, and effector–effector interactions shape virulence networks, while the cumulative burden of ETI-eliciting effectors generates a quantitative ETI load that constrains pathogen fitness and informs strategies for durable disease resistance.The authors synthesize recent developments in effector-mediated host-microbe interactions into an interconnected, systems-level view of phytobacterial effectoromes, in which functional redundancy, low target specificity, and effector–effector interactions shape virulence networks, while the cumulative burden of ETI-eliciting effectors generates a quantitative ETI load that constrains pathogen fitness and informs strategies for durable disease resistance.

## Introduction

Phytopathogenic bacteria are a serious threat to crop production and global food security (Savary et al. 2019). The development and deployment of disease-resistant cultivars is an essential strategy to mitigate these losses and ensure sufficient agricultural productivity to support the growing global population. These crop development strategies are increasing reliant on a detailed understanding of plant immunity, which detects and responds to invading pathogens through sophisticated, interconnected, dual-tiered immune responses: pattern-triggered immunity (PTI) and effector-triggered immunity (ETI) (Jones and Dangl 2006, Nelson et al. 2018, Yuan et al. 2021b, Ngou et al. 2021, Pruitt et al. 2021, Tian et al. 2021, Ngou et al. 2022, Zhao et al. 2022).

Phytopathogens use a variety of means to overcome host defenses and promote their own fitness during the infection process. One of the most important mechanisms used by bacterial pathogens is the type III secretion system and its associated effectors. These effectors are extremely diverse and show a high level of presence/absence heterogeneity even among closely related strains. For example, the phytopathogen *Pseudomonas syringae* has ∼70 distinct families of effectors of varying size (range 1–362 unique alleles, mean 73 ± 10.5 (stderr)), while each strain carries an average of 30 ± 10.1 (stderr) effectors (Dillon et al. 2019). These effectors are translocated directly into the host cell where they attempt to suppress the immune response and disrupt cellular homeostasis. The totality of all effectors carried by a pathogen species is called the effectorome, while the specific collection of effectors carried by any one strain is called the strain’s effector repertoire, suite, or arsenal.

While effectors are necessary for pathogen fitness, they can also be directly or indirectly recognized by host nucleotide-binding, leucine-rich repeat immune receptors (NLRs). The interaction between host NLRs and pathogen effectors is very specific in that specific alleles in individual NLR families recognize specific alleles in individual effector families. If recognition occurs, an ETI will be mounted by the plant, effectively stopping most pathogen outbreaks.

Not surprisingly, the plant immune system is also complex and multi-tiered. The first tier of plant immunity involves plasma membrane-localized pattern recognition receptors (PRRs) that perceive conserved pathogen-associated molecular patterns (PAMPs), such as conserved regions of the bacterial flagellin protein, initiating PTI responses. The second tier of plant immunity employs intracellular NLRs responsible for the ETI response. Recent advances have deepened our understanding of the downstream signaling mechanisms of NLR activation (Ngou et al. 2022, Huang et al. 2023). NLRs are phylogenetically classified into two major groups based on N-terminal domains: TIR-NLRs (TNLs; containing Toll/interleukin-1 receptor domains) and CC-NLRs (CNLs; containing coiled-coil domains), each activating distinct signaling cascades. Structural studies reveal that CNL-type receptors, such as ZAR1 and Sr35, form conserved homopentameric resistosomes upon activation. These complexes exhibit Ca+ channel activity, and trigger an increase in intracellular Ca+ concentration followed by immune activation and cell death (Wang et al. 2019, Bi et al. 2021, Förderer et al. 2022). In contrast to CNLs, TNLs assemble into homotetrameric complexes endowed with NADase activity (Wan et al. 2019). These complexes metabolize NAD + and ATP, generating secondary messengers that exhibit pathway-specific functions. For example, pRib-AMP specifically promotes the assembly of the ADR1-EDS1-PAD4 complex to activate ADR1, whereas ADPr-ATP drives the formation of the NRG1-EDS1-SAG101 complex to activate NRG1 (Huang et al. 2022, Jia et al. 2022). The activated helper NLRs (ADR1 or NRG1) subsequently oligomerize into calcium ion channels (Yu et al. 2024b, Jacob et al. 2021, Wu et al. 2024, Huang et al. 2025, Xiao et al. 2025), engaging partially redundant yet distinct immune signaling modules (Chhillar et al. 2025). The synergistic activation of ETI and PTI generates amplified immune responses characterized by sustained reactive oxygen species (ROS) bursts, callose deposition, and localized hypersensitive cell death (Yuan et al. 2021a, Nelson et al. 2018, Ngou et al. 2021, Pruitt et al. 2021, Tian et al. 2021, Zhao et al. 2022). This synergistic interaction between the two immune layers enables significant augmentation of signaling amplitude and system robustness, creating an integrated defense network that effectively restricts pathogen invasion at multiple infection stages while suppressing disease symptom development.

The predominant focus in effector biology research has centered on investigating the virulence or ETI-elicitation functions of individual effectors, while studies at the level of effectoromes remains an emerging field (Wang et al. 2022, Cai et al. 2023). However, there is increasing evidence that effectors function not as isolated entities but in coordinated and interdependent ways that can best be characterized by an interactome network (Bundalovic-Torma et al. 2022, Sanchez-Garrido et al. 2022). In this review, we propose that effector functional redundancy, multifunctionality, and interdependence result in interconnected effectorome networks, where individual network nodes represent plant cellular and physiological processes targeted by functionally redundant effectors, i.e. functional modules. This framework changes the focus from the classical gene-for-gene model to a systems-level NLRome-effectorome interface model. In this model, plant NLRomes combinatorially surveil pathogen effectoromes. Specific infection outcomes depend on the range of NLRs carried by the individual host plant and the effectors carried by the pathogen strain. If pathogen strains carry multiple ETI-eliciting effectors, the host’s ETI response may be quantitatively enhanced. The total burden of ETI-eliciting effectors carried by a strain can be thought of as the strain’s ETI-load. To counter this ETI load, pathogens have evolved effectors that are able to suppress specific ETI responses. The equilibrium between the quantitative ETI response and the ETI-suppression capacity of the pathogen largely determines the outcome of the interaction, i.e. disease or immunity. To enhance crop resistance, engineering strategies must amplify ETI-load while reducing ETI-suppression. This necessitates prioritizing NLRs that target effectors that play a critical role in the disease process, are evolutionarily conserved and broadly distributed, and which have high ETI-suppression activity. Rational stacking of NLR genes targeting such effectors in crop genomes presents an exciting approach for establishing durable disease resistance.

Box 1: **Quantitative ETI**: In the plant-pathogen interaction system, quantitative ETI responses constitute quantitative variations arising from the dynamic interplay between effectoromes and the NLRome. Rooted in the well-established qualitative ETI framework, specifically the gene-for-gene model based on Avr-NLR recognition, this concept retains that core mechanism while integrating quantitative determinants such as the abundance of effectors and NLR proteins, the affinity of their interaction, the strength of PTI signaling, and the suppression of ETI responses by effectors. Crucially, the proposal of quantitative ETI does not overturn or replace the classic gene-for-gene hypothesis; rather, it is an extension built upon this foundation and enhances its general applicability.

**Effector Redundancy:** At the phenotypic level, effector redundancy manifests as the absence of significant alteration in pathogenicity upon deletion of one or a few effectors. At the molecular mechanistic level, it is characterized by overlapping functions among a subset of effectors, including those targeting the same host target with identical biochemical activity, those targeting the same host target with distinct biochemical activities, those targeting different targets of the same host pathway, or those targeting different pathways of the same host process, etc. Functional redundancy of effectors does not imply functional superfluity; rather, it is essential for pathogens to maintain pathogenic robustness, efficacy, and resilience.

**Effector Multifunctionality:** Refers to the capacity of effectors to simultaneously target multiple host targets residing in distinct pathways and modulate diverse physiological processes of the host. Multifunctionality represents a common attribute of effectors, yet this is not absolute; there may exist effectors endowed with a singular yet critical function.

**Effector Network:** Pathogen effectors are not isolated entities but engage in extensive interactions, ultimately forming an integrated networked system. First, redundant effectors constitute effector modules that target the same host process. Moreover, host processes are extensively interconnected, and the multifunctionality of effectors enables them to reside in multiple modules simultaneously, thereby facilitating the integration of distinct effector modules into a cohesive effector network. Therefore, we refer to this network as the “interconnected module model”.

## Effector modules target specific host processes

Functional redundancy constitutes a hallmark feature of pathogenic bacterial effectoromes. This phenomenon is most clearly seen when pathogen fitness and virulence capacity are maintained after the removal of one or more effectors from a strain’s effector repertoire. This trait is well-documented across taxonomically diverse pathogens, including phytopathogens *P. syringae* (Chakravarthy et al. 2018), *Ralstonia solanacearum* (Lei et al. 2020), and *Xanthomonas oryzae* pv. *oryzae* (Yan et al. 2023), and zoonotic pathogens *Citrobacter rodentium* (Ruano-Gallego et al. 2021) and *Salmonella Typhimurium* (Chen et al. 2021). Experimental validation through systematic effector knockout demonstrates remarkable functional resilience. For example, the mouse pathogen *C. rodentium* retains full pathogenicity even after the deletion of 60% (24/31) of its repertoire (Ruano-Gallego et al. 2021). Minimal effectoromes comprising ∼30% (8/28) and ∼17% (5/30) of effectors have been shown to be sufficient for host colonization and disease progression in *P. syringae* (Cunnac et al. 2011) and *S. Typhimurium* (Chen et al. 2021), respectively. Critically, this redundancy reflects likely evolutionary necessity rather than biological excess (Ruano-Gallego et al. 2021). Chen and colleagues (Chen et al. 2021) identified a minimal set of five *Salmonella* Typhimurium SPI-2 effectors that enabled replication and survival within host cells under controlled laboratory conditions; however, this minimal effector repertoire was insufficient to cause systemic disease in susceptible hosts, was rapidly cleared in antibiotic-altered environments, and was unable to colonize genetically resistant hosts. Hence, functional redundancy of effectors reveals a “context-dependent essentiality” that underscores its critical role in sustaining virulence robustness (Ruano-Gallego et al. 2021).

**Figure 1:**
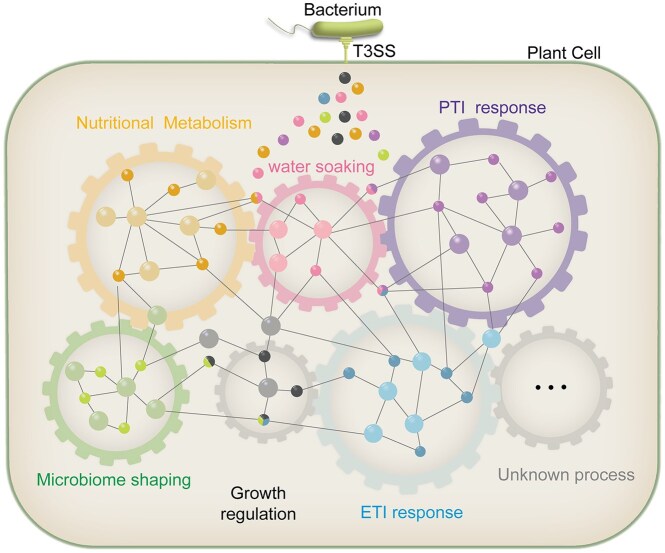
Type III effectors form an interconnected modular network *in vivo*. To establish infection, pathogens require coordinated manipulation of multiple host physiological processes (represented by colored gears), which are intrinsically interconnected (depicted by interlocking gears). Each module comprises a group of effectors (small circles) targeting critical host components (big circles) within a specific process, with intra-module effector interactions (buffering or synergism) ensuring robust and efficient manipulation. Host physiological interdependence (gear teeth interlock) and multifunctional effectors (split-colored circles spanning adjacent gears) jointly drive inter-module connectivity. Notably, effectors in any module may trigger NLR-mediated ETI responses, yet their virulence persistence relies on counteraction by dedicated ETI-suppression effector modules, thereby reinforcing cross-module network integration.

Our current understanding of effectorome robustness suggests a modular architecture in which pathogen effectors are organized into functional modules that execute core pathogenic strategies, including but not limited to PTI and/or ETI suppression (Wang et al. 2022), nutrient acquisition (Cai et al. 2023), water-soaking (Xin et al. 2016), and microbiome manipulation (Wu et al. 2019) (Fig. 1). Each functional module includes multiple effector families that have convergently evolved to target the same host process. Each targeted process can be thought of as an interaction node. While effectors within a module converge on the same process, the mechanisms of this targeting may vary and include: focused disruption of specific complexes; serial attacks along a signaling cascade; and/or parallel attacks across coordinated pathways. The functional redundancy embedded within modules provides robustness, so that the loss of one or more effectors can be compensated for by the remaining effectors within the same functional module, thereby ensuring the pathogen effector—host process interaction is robust and resilient. This robustness not only works on an ecological timeframe, e.g. if a mobile genetic element disrupts a key effector in a particular strain, but also on an evolutionary timeframe, e.g. if the host acquires a mutation in an effector target that disrupts the effector function. This network paradigm is in accord with earlier observations of redundant effector groups (Kvitko et al. 2009) and effector guilds (Bundalovic-Torma et al. 2022). A recent systematic review has found that animals also recognize effectors via a “detection of impaired function” model analogous to the plant’s “decoy or guard” model (Remick et al. 2023), and restrict bacterial infection through programmed cell death and immune activation. Such parallels in recognition models and consequences suggest that animal pathogen effector repertoires may face similar selective pressures as those in plants, ultimately leading to shared features like effector redundancy.

A prime illustration of functional redundancy within an effector module can be seen in the suppression of plant PTI responses, where *P. syringae* effectors target this pathway in a variety of ways that span the signaling cascade. Initially, membrane-localized sabotage occurs through transcriptional inhibition of FLS2 receptors by HopU1/HopQ1, coupled with AvrPtoB-mediated proteasomal degradation of existing receptors, effectively eradicating pathogen recognition capacity (Xiang et al. 2008, Nicaise et al. 2013, Hann et al. 2014). As immune signals attempt to propagate into the cytoplasm, HopZ1a-mediated acetylation of receptor-like cytoplasmic kinases (RLCKs) synergizes with AvrPphB’s proteolytic cleavage of these signaling molecules to terminate signal transduction (Zhang et al. 2010, Bastedo et al. 2019). Subsequently, HopAI1 and AvrRpt2 suppress the phosphorylation activation of MAPKs, thereby inhibiting the cascade amplification of immune signals (Eschen-Lippold et al. 2016a, Zhang et al. 2007). The targeting of one or more components of the PTI signaling network may sufficiently compromise the process to block the activation of nuclear defense programs, as evidenced by inhibited nuclear translocation and activation of NPR1 transcription factors (Chen et al. 2017). The effectors of the PTI-suppression module target multiple stages of PTI responses in a convergent manner, ensuring robust and resilient immune suppression. This modularity paradigm extends beyond PTI suppression. Pathogens have also evolved effector modules targeting other physiological processes, such as the ubiquitin-proteasome system (Ramachandran et al. 2021), autophagy (Lal et al. 2020), endomembrane transport and homeostasis (Jeon and Segonzac 2023), mitochondrial function (Nandi et al. 2021), and others.

## Interconnected host processes link effector modules into an interaction network

Effector modules are primarily delineated based on distinct host physiological processes, while host physiological processes are dynamically interconnected at the molecular, cellular, and physiological levels, thereby fostering extensive interconnections among effector modules. For example, Tsuda et al. (2009) examined a modular signaling network where salicylic acid (SA), jasmonic acid (JA), ethylene (ET), and PAD4 modules operate via compensatory and synergistic interactions to ensure robust and efficient immune responses. Building upon this, Kim et al. (2014) established a PTI signaling model comprising four sectors (JA, SA, ET, PAD4), demonstrating that PAMPs directly stimulate JA, ET, and PAD4 sectors, while the SA sector acts downstream to regulate the immune response, while ET enhances network robustness by suppressing JA and PAD4 sectors, and collectively forming a quadripartite switch that systemically guarantees immune robustness. Beyond these signaling architectures, further examples of interconnected host processes include the regulatory cascade where stomatal aperture modulates water-soaked lesion formation while subsequently influencing immune activation and nutrient efflux (El Kasmi et al. 2018, Hu et al. 2022, Yao et al. 2023); the delicate equilibrium between developmental processes and immune responses in plants (Zhang et al. 2020), and so on (Iqbal et al. 2021, Hou et al. 2024).

Given the integration of host processes, it follows that effector modules should also exhibit extensive interconnections (Fig. 1). The most common and simplest example of how this is achieved is through effector multifunctionally, in which individual effectors have the capacity to engage multiple host targets across distinct physiological processes. Effectors frequently exhibit broad target promiscuity rather than strict specificity, in other words, they act more like shotguns than sniper rifles (Bundalovic-Torma et al. 2022). This promiscuity is presumably selectively beneficial since effectors with broad target specificities would retain their utility even if one of their targets evolved out of the effector specificity spectrum. In contrast, effectors with strict target specificities would not contribute to pathogen fitness if the host target evolved out of the effector specificity spectrum. An example of this multifunctionality is observed in *P. syringae* AvrRpt2b effector, which operates as a cysteine protease to suppress PTI through both RIN4 cleavage (Lim and Kunkel 2004) and MAPK pathway inhibition (Eschen-Lippold et al. 2016b); while paradoxically enhancing pathogenesis via auxin/IAA protein turnover-mediated activation of indole-3-acetic acid signaling (Cui et al. 2013). Similarly, the E3 ubiquitin ligase AvrPtoB demonstrates polyvalent activity through coordinated degradation of FLS2 (PTI suppression) (Göhre et al. 2008), ADR1 homologs (ETI suppression) (Wang et al. 2023), and ATG1 phosphorylation blockade (autophagy inhibition) (Lal et al. 2020). The *R. solanacearum* effector RipAC epitomizes spatial multifunctionality by targeting PUB4 ubiquitin ligase to modulate BIK1 homeostasis (PTI suppression) (Yu et al. 2022b), while concurrently inhibiting MAPK-mediated SGT1 phosphorylation (ETI suppression) (Yu et al. 2020), and altering root architecture to facilitate bacterial invasion (Yu et al. 2024a). The multifunctional nature of effectors has been substantiated not only through mechanistic studies of individual effectors, but also corroborated by effectorome-level investigations. This functional versatility is exemplified by 83 effectors from bacterial, fungal, and oomycete pathogens that collectively target 843 *A. thaliana* proteins, demonstrating the prevalent phenomenon of single effectors engaging multiple host targets (Mukhtar et al. 2011, Weßling et al. 2014). Such pleiotropic targeting enables individual effectors to functionally bridge multiple modules, thereby establishing extensive interconnections between distinct modules.

How is effector multifunctionality achieved? One potential hypothesis is that: In many cases, enzyme promiscuity and specificity are related to the flexibility of their conformation. For example, ancestral β-lactamases possess flexible conformations that mediate the degradation of multiple β-lactam antibiotics, whereas modern β-lactamases exhibit greater structural rigidity and are capable of degrading only penicillin (Zou et al. 2015). A similar case is observed in trypsin and caspase (Hedstrom 1996, Exconde et al. 2024), indicating that conformational flexibility is often associated with the promiscuity of enzymatic catalytic capacity. Effectors are typically mechanically labile—a property that facilitates their unfolding to passage through the narrow needle of the T3SS (LeBlanc et al. 2021). This structural instability of effectors may increase the flexibility of interfaces mediating protein–protein interactions or substrate recognition, thereby enabling them to simultaneously target multiple host targets and exhibit multifunctionality. Effector multifunctionality may be the result of selection for low substrate specificity. As discussed above, this promiscuity has clear selective benefits for the pathogen but may also benefit the host when the effector interacts with a decoy protein monitored by NLR receptors. This effector-decoy interaction is also an evolutionarily robust strategy for the host since it hijacks the selective forces driving virulence evolution (Cesari 2018, Bundalovic-Torma et al. 2022).

## Effector evasion and suppression enable robust and resilient counters to NLR recognition

The first study of the scope of ETI elicited by a full pathogen effectorome (i.e. the ETI landscape) found that nearly all (96.8%) of *P. syringae* strains carry at least one effector homolog that has the potential to elicit ETI in *A. thaliana* ecotype Col-0 (Laflamme et al. 2020). This was a surprising discovery since nearly all the strains in the study collection were isolated as pathogens; leading to the obvious question, how do strains maintain their pathogenic potential when faced with a potential large ETI-load? Pathogenic strains must have some means to evade or suppress the ETI response.

Genetically, ETI evasion can be achieved via coding sequence diversification, gene pseudogenization, or transcriptional modulation of individual ETI-eliciting effector genes (Wang et al. 2022). For example, *P. syringae* pv. *phaseolicola* 1302A genomically excises and supercoils the 106 kb genomic island PPHGI-1 containing AvrPphB (syn. HopAR1) to evade the detection by the bean R3 NLR (Godfrey et al. 2011, Neale et al. 2018). Similarly, *P. syringae* pv. *tomato* DC3000 carries a truncated HopO1c that does not elicit ZAR1-mediated ETI in *Arabidopsis* (Laflamme et al. 2020). However, the dual function of effectors as virulence factors and potential ETI-elicitors means that ETI evasion may carry the risk of compromised virulence. Since most mechanisms of ETI evasion involve some form of effector functional loss, there must also be a complementary mechanism that preserves essential effector virulence functions (Rufián et al. 2023). Effector functional redundancy is the simplest means to generate an evolutionary buffer that enhances pathogen robustness and resilience in the face of these selective pressures (Ghosh and O’Connor 2017).

While ETI evasion generally occurs through well understood evolutionary mechanisms acting on individual loci, recent work has demonstrated that ETI suppression is likely achieved through effector–effector interactions, i.e. an integrated response of the effectorome (Martel et al. 2022). Martel and colleagues demonstrated the importance of ETI suppression when studying the ETI-eliciting potential effectors in different genetic backgrounds (Martel et al. 2022). They found that the *P. syringae* effectors AvrPto1 and HopT1 elicited ETI when expressed in the *P. syringae* strain PmaES4326 but not when expressed in PtoDC3000. Using an ETI-suppression in PmaES4326, they found that AvrPto1-mediated ETI was suppressed in the PtoDC3000 background by the HopQ1 effector, while the HopT1-mediated ETI was suppressed by the effectors HopG1 and HopF1.

**Figure 2:**
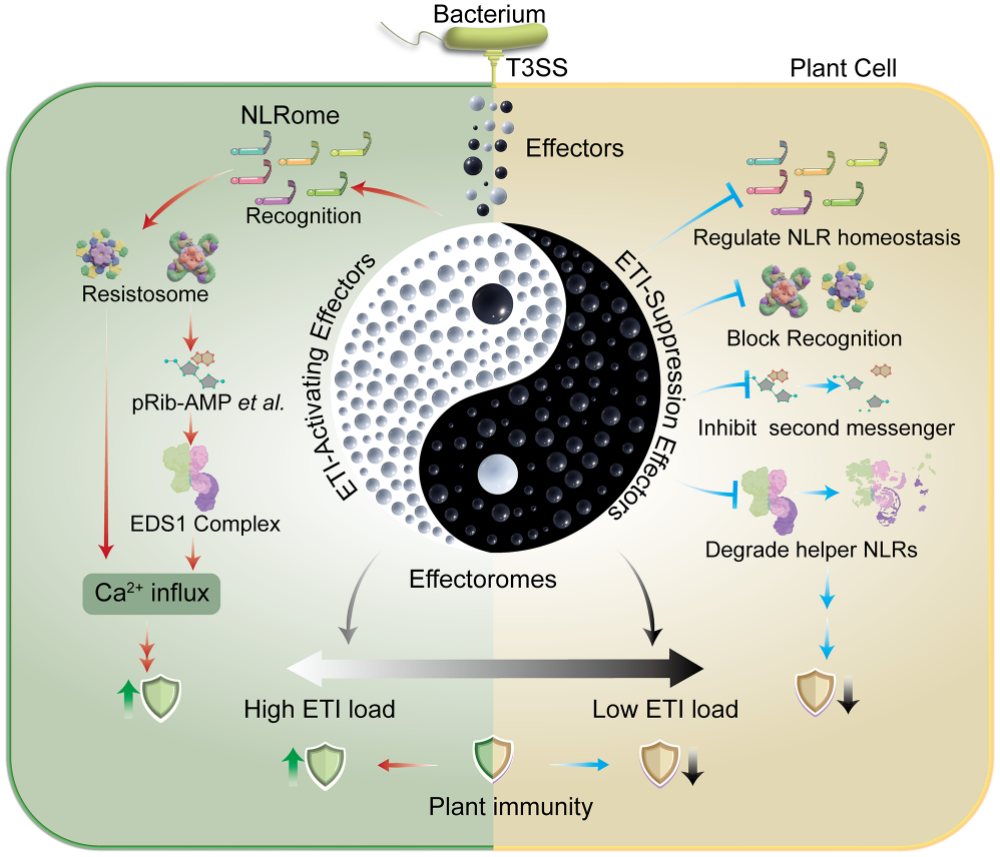
Plant immune output is determined by the dynamic equilibrium of ETI load and ETI-suppression through pathogen effectorome-plant NLRome interactions. A Taiji (Yin-Yang) model illustrating the dynamic interplay between pathogen effectoromes and plant ETI (effector-triggered immunity) and the balance of opposing yet interdependent forces governing pathogen-host interactions. Pathogen effectoromes include effectors that may be either ETI-activating (Yang, where black prevails) or ETI-suppressing (Yin, where white prevails). The balance between activation-suppression dictates infection outcomes: ETI activation dominance causes high ETI load and failed infection, while suppression dominance leads to low ETI load and successful infection; in compatible interactions, they counterbalance to yield a “zero” neutral phenotype. The subsets have fuzzy, dynamic boundaries (curved line, not straight), and mutual inclusion—exemplified by AvrPtoB, which both activates and suppresses ETI (white/black spheres in opposite regions). These subsets shift with pathogen/host changes or evolution, even if partners are fixed. The Taiji diagram’s two sides illustrate molecular mechanisms: Left (ETI activation): The NLRome recognizes ETI-activating effectors, forming CNL or TNL resistosomes. CNL resistosomes directly form Ca^2+^ channels to induce immunity; TNL resistosomes use NADase activity to generate second messengers (e.g. pRib-AMP), triggering EDS1-PAD4-ADR1/EDS1-SAG101-NRG1 complexes that activate helper NLRs and Ca^2+^ channels. Right (ETI suppression): ETI-suppressing effectors target ETI steps—regulating NLR proteostasis, blocking recognition, degrading second messengers/EDS1 components—to inhibit immunity and promote infection.

The examples above describe individual effectors specifically suppressing the ETI responses elicited by other effectors, but this is not necessarily the only possible outcome. ETI suppression can be classified into two categories based on target specificity; specific suppression and broad-spectrum suppression (Fig. 2). Specific ETI suppression entails the targeted preventing of one or a few ETI responses by, typically, interfering with the effector recognition processes (Ray et al. 2019). For example, the *P. syringae* effector AvrRpm1 promotes the phosphorylation modification of the *A. thaliana* RIN4 decoy, which is detected by the guardee NLR RPM1, thereby activating the ETI response (Mackey et al. 2002, Redditt et al. 2019). Another effector with cysteine protease activity, AvrRpt2, promotes the cleavage and degradation of RIN4, thus inhibiting RPM1-mediated ETI (Coaker et al. 2005). Thus, AvrRpt2 can be considered a specific ETI suppressing effector. Yet, the interaction becomes even more interesting because *A. thaliana* has “upped the ante” with another NLR receptor, RPS2, which senses the degradation of RIN4 by AvrRpt2 and activates the ETI response (Axtell et al. 2003, Li et al. 2014). HopF2 is employed to inhibit the degradation of RIN4 by AvrRpt2, thereby specifically suppressing the ETI it induces (Wilton et al. 2010). Such specific and multi-layered NLR-effector interplay epitomizes the coevolutionary dynamics conceptualized in the ZigZag model (Jones and Dangl 2006).

The second category of broad-spectrum ETI suppression entails the systemic disruption of convergent ETI signaling nodes, typically targeting key second messengers, their receptors, and downstream activators. Examples include: the *Xanthomonas* XopQ and *Phytophthora* Avr3B effectors through their 2’,3’-cAMP/cGMP phosphodiesterase activity, which hydrolyzes TIR-NLR-derived immunomodulatory nucleotides (Yu et al. 2022a), and *P. syringae* HopAM1 effector which subverts immune signaling by generating non-canonical nucleotide analogs that competitively inhibit TIR-NLR signaling cascades (Eastman et al. 2022, Manik et al. 2022, Hulin et al. 2023). A distinct suppression mechanism involves destabilizing helper NLR complexes essential for signal amplification. The *P. syringae* AvrPtoB effector employs E3 ubiquitin ligase activity to degrade ADR1-family helper NLRs, thereby broadly suppressing ETI triggered by diverse effectors including HopAD1, HopT1, and HopAA (Jamir et al. 2004, Wei et al. 2015, Gimenez-Ibanez et al. 2018, Wang et al. 2023). The *Ralstonia solanacearum* effector RipAC disrupts NLR protein homeostasis by targeting the NLR-associated chaperone SGT1, while also suppressing cell death triggered by diverse effectors including RipAA, RipE1, and others (Takahashi et al. 2003, Yu et al. 2020, Nakano et al. 2021). Notably, broad-spectrum suppressors face evolutionary constraints through NLR surveillance systems that detect their immunosuppressive activity, such as the recognition of AvrPtoB by the tomato Pto (Salmeron et al. 1996) and the *A. thaliana* SNC1 (Wang et al. 2023). These interactions epitomize the complex infection dynamics arising when effectors harbor both immune activation and suppression capacities.

## Effectorome-NLRome interactions result in quantitative ETI responses

The classical gene-for-gene model describes discrete infection outcomes (i.e. immunity or disease) governed by one-on-one interactions between effectors and their cognate NLR receptors; in other words, a qualitative Mendelian trait under monogenic control (Flor 1942). This gene-for-gene framework was originally considered to be distinct from quantitative disease resistance, or what has been known as partial disease resistance since at least the 1930s (Torrie 1939, Poland et al. 2009). Nevertheless, our current understanding of the mechanistic basis of plant immunity and the significance of population-level genetic diversity makes it difficult to maintain the distinction between gene-for-gene complete resistance and quantitative partial resistance (Yuan et al. 2021b, Yu et al. 2024c, Lu and Tsuda 2021, Nabi et al. 2024).

Current studies reveal that the protein dosage of certain ETI-inducing effectors plays a pivotal role in determining immune outputs, prompting reconsideration of the quantitative properties of ETI. For instance, the *P. syringae* DC3000 effector AvrPtoB is detected by the NLR receptor SNC1, leading to its oligomerization and activation (Wang et al. 2023). Although this recognition event occurs, the *snc1* knockout mutant exhibits no susceptibility compared to wild-type plants when inoculated with *P. syringae* DC3000 at an optical density (OD₆₀₀ = 0.0001, 10⁶ colony-forming units [CFU]/ml) (Yang and Hua 2004). However, at a higher inoculation concentration (OD₆₀₀ = 0.02), *P. syringae* DC3000 carrying AvrPtoB grows significantly better in the *snc1* mutant than in wild-type plants, indicating that recognition of AvrPtoB enhances disease resistance (Wang et al. 2023). When the inoculum concentration is further increased to OD₆₀₀ = 0.3, AvrPtoB-expressing *P. syringae* D36E strains induces upregulation of the *PR1* gene in wild-type *A. thaliana*, an effect abolished in the *snc1* mutant (Wang et al. 2023). This elevated inoculum likely correlates with increased intracellular AvrPtoB abundance, as in situ visualization of effector delivery during natural infection shows multiple pathogens can secrete effectors into the same host cell, with effector abundance often scaling with inoculum concentration (Park et al. 2017). The case of AvrPtoB is not isolated. The *P. syringae* effector AvrE is recognized by CAR1 in *A. thaliana*, yet AvrE expressed from identical promoters via plasmid or chromosomal integration induces varying degrees of immune protection in hosts, suggesting differential expression levels drive distinct immune outcomes (Laflamme et al. 2020). Similarly, the *R. solanacearum* effector RipE1 is ubiquitinated and degraded in hosts but evades this via phosphorylation or interaction with ubiquitin proteases to enhance stability. A phosphomimetic mutant of RipE1 accumulates to higher levels in hosts (Yu et al. 2025) and induces stronger cell death in *N. benthamiana* (data not shown). Notably, estradiol-inducible expression of CC-NLR-recognized effectors in *A. thaliana* triggers cell death; reducing estradiol concentration to weaken effector expression diminishes or eliminates this phenotype. Interestingly, the absence of cell death does not imply failed ETI activation, as concurrent activation with PTI still induces cell death (Ngou et al. 2021). It is worth noting that sublethal effector concentrations may partially activate ETI without causing cell death, implying cell death occurs only when immune activation exceeds a threshold—though a comprehensive understanding of this threshold remains lacking (Ngou et al. 2021). In summary, these pioneering examples collectively suggest that varying effector dosages elicit distinct ETI responses, supporting the quantitative hypothesis of ETI.

Effector-NLR interactions can give rise to quantitative ETI responses via a number of mechanisms. The PTI-ETI crosstalk model may further support this quantitative hypothesis of ETI responses. In this model, ETI-induced ROS bursts and pathogen restriction are significantly attenuated or even abolished when PTI is compromised; only the co-activation of PTI and ETI leads to stronger immune activation, cell death, and pathogen restriction compared to PTI alone (Yuan et al. 2021a, Ngou et al. 2021). Here, ETI activation is partially viewed as an amplification of PTI responses, a classic quantitative response typically characterized by metrics such as ROS burst intensity, callose deposition levels, transcription of immune marker genes, and bacterial proliferation rates. Notably, recent evidence for the intrinsic uncoupling of cell death and immune activation in ETI responses further supports the notion that ETI is at least partially quantitative (Bendahmane et al. 1999, Chhillar et al. (Chhillar, Nguyen and Yeh 2025)).

While cell death is a strictly binary process at the single-cell level (i.e. occurrence or non-occurrence), it exhibits quantitative characteristics at both the molecular and tissue levels. For instance, at the molecular level, an ETI signaling cascade initiated via TIR resistosome activation involves NADase enzymatic activity, generation of secondary messengers, calcium channel formation, and Ca^2+^ influx mediated by helper NLRs (Wan et al. 2019, Wang et al. 2019, Bi et al. 2021, Jacob et al. 2021, Huang et al. 2022, Jia et al. 2022). Key signaling processes within this cascade—including NADase activity, abundance of secondary messengers, and calcium ion flux—are quantitative in nature. At the tissue level, cell death is also quantified using metrics such as the hypersensitive response (HR) index (integrating necrosis severity, area, and frequency) and ion leakage rates. Collectively, these observations suggest that cell death, a seemingly binary biological process, is not a purely binary process; rather, its internal dynamics typically involve quantitative changes giving rise to qualitative transformations.

Another factor influencing the quantitative ETI responses is natural genetic diversity of pathogen effectors and plant immune components. Different alleles of an NLR may recognize an effector with different binding affinities or efficiencies, leading to stronger or weaker immune responses. For example, the NLR proteins Pikm1/Pikm2 recognize the *Magnaporthe oryzae* effector AvrPikD to trigger intense cell death, while recognition of its homologous effectors AvrPikE and AvrPikA results in a reduced degree of cell death (measured by the HR index), and they fail to recognize other homologous effectors such as AvrPikB, AvrPikC, and AvrPikF. Pikm1 mediates this recognition primarily through its interaction with AvrPik effectors via the heavy metal-associated (HMA) domain; mutations of a few amino acids in the HMA domain are sufficient to enable it to recognize all AvrPik family effectors. Furthermore, protein–protein interaction studies have revealed that the presence or absence of interaction capability between Pikm1 and AvrPik effectors, and even the degree of interaction strength, determines the presence/absence and intensity of cell death resulting from their interaction (De la Concepcion et al. 2018, Zhu et al. 2025). Iakovidis and colleagues (Iakovidis et al. 2016) showed variation in the disease response of different *A. thaliana* ecotypes to the *P. syringae* effector HopAM1. Interestingly, these differences were not tied to pathogen growth, but rather to how strongly the immune response was activated and virulence (i.e. host damage). They treated both cell death and chlorosis as quantitative traits in a genome-wide association study to identify multiple genetic loci that had varying effects on the ETI outcome. Such quantitative changes in ETI responses may also involve multiple mechanisms. For example, differences in NLR gene expression levels or receptor abundance, or NLR alleles differing in their N-terminal domains, could all contribute to the quantitative modulation of ETI responses.

Quantitative ETI responses are also a consequence of interactions between multiple functional modules of the host. Katagiri and colleagues showed that immune signaling cascades result from interactions among modules predominantly governed by JA, ET, SA, and PAD4 (Tsuda et al. 2009, Kim et al. 2014, Hillmer et al. 2017). Similarly, Delplace et al. (2020) demonstrate that quantitative disease resistance to *Xanthomonas campestris* in *A. thaliana* is controlled by an immune network centered around the kinase-like protein RKS1 (syn ZRK1). RKS1 physically interacts with proteins in five functional modules: receptor signaling/regulation; protein metabolism; small-molecule metabolism; vesicle-mediated transport; and regulatory processes. This modular architecture is highly decentralized, with no single node acting as a central hub. The authors used single and double gene knockout to show functional redundancy and buffering among the modules conferred robustness to genetic perturbations (Chauveau and Roby 2024, Delplace et al. 2024). The interconnections between these functional modules associated with activation and response pathways results in an interaction network that influences the quantitative magnitude of the ETI response.

A less studied factor influencing the quantitative nature of ETI is the degree to which ETI suppressing effectors are able compensate for the ETI load carried by a pathogen strain (Martel et al. 2022). We are only beginning to understand how effector–effector interactions impact the ETI response, and consequently, host-pathogen interactions. The strongest argument for this is the previously discussed finding that nearly every *P. syringae* strain carries a homolog of an ETI-eliciting effector, yet most of these strains were isolated as pathogens, raising the question of how ETI-eliciting effectors can be so prevalent in a pathogen species known to cause so much disease? As discussed, one potential answer is that the ETI-elicitation is suppressed by other effectors. Other notable examples beyond the ones discussed previously include the *P. syringae* AvrPtoB (syn HopAB1) and *R. solanacearum* RipAC effectors, which are highly prevalent effectors with broad-spectrum suppression of NLR-mediated defenses through ADR1 degradation (Wei et al. 2015, Wang et al. 2023) and SGT1 phosphorylation blockade (Yu et al. 2020, Nakano et al. 2021), respectively. Effectors capable of suppressing ETI appear to be widespread within pathogen effectoromes, thereby constituting an active and prevalent mechanism for reducing ETI load (Fig. 2).

It is worth noting that the concept of quantitative ETI does not negate or replace the classic gene-for-gene hypothesis, but rather represents an extension built upon its fundamental principles. The quantitative ETI framework posits that plant disease resistance is not determined solely by NLR protein recognition of effector proteins. Instead, the various factors mentioned above exert quantitative influences on this recognition event and the subsequent signal transduction, consequently leading to variations in the degree of resistance. In host-pathogen interactions characterized by complete or high resistance, NLR recognition of Avr proteins triggers an immune response of sufficient magnitude that the impact of other factors (e.g. ETI-suppressing effectors) is minimal or negligible; under these circumstances, the ETI response aligns with the qualitative trait described by the gene-for-gene hypothesis. Conversely, in interactions involving intermediate resistance—situated between fully resistant and susceptible hosts—both NLR-Avr recognition and a multitude of other factors must be comprehensively considered, resulting in a quantitative ETI response. This latter model is also particularly applicable to more complex interaction systems, such as those involving effectorome–NLRome interactions.

The effectorome-NLRome interaction model proposed in this review is primarily based on plant pathogenic bacteria. Other pathogens, including fungi, oomycetes, etc., despite having significantly different lifestyles compared to bacteria, have effectors that interact with the host plant’s unified PTI-ETI immune system and coevolve through a similar dynamic ZigZag model. This suggests that extending this model to other pathogens is possible, but the precise extension of this model requires further careful verification.

## Systems-based approaches to study effectorome-NLRome interactions

The interaction of a strain’s effector repertoire with its hosts suite of immune receptors largely determines the outcome of individual infection. Likewise, the species-wide effectorome and host NLRome determine the global scope of ETI activation and suppression and shape the dynamics of the co-evolutionary arms race between these species (Fig. 2). There are clear and obvious fitness costs when an organism, or species as a whole, lacks an effector or NLR required to counter the other, but there are also fitness costs associated with carrying effectors or NLRs in the absence of their corresponding interactor (Tian et al. 2003). Despite the complexities of these systems and the challenges associated with characterizing the functional features and underlying mechanisms of effectorome-NLRome interactions, a number of promising approaches have emerged that hold significant potential. For example, Ruiz-Bedoya and colleagues (Ruiz-Bedoya et al. 2023) made expression clones of all 36 effectors carried by the *A. thaliana* pathogen *P. syringae* DC3000, and then transformed each into an avirulent DC3000-derivative that has knockouts of all its effectors, to make a library of 36 strains that carry only one effector. They first confirmed that multiple effectors are required for virulence by performing growth assays with each effector clone individually and found no significant growth on *A. thaliana*. They then asked if a “metaclone” population of 35 effector clones (one effector was removed because it elicits ETI when expressed on a multicopy plasmid) was able to cause virulence similar to the wildtype DC3000 strain. Remarkably, the wildtype and metaclone population grew to the same level. This showed that collective virulence could emerge in a population of individually avirulent clones if those clones share the benefits of their secreted effectors, i.e. effectors are public goods. They also showed that adding one ETI-eliciting effector into the metaclone would reduce the fitness of the entire metaclone population. This metaclone approach holds great potential for identifying functional modules and dissecting effector–effector interactions, since it allows the mixing and matching of any number or combination of effectors.

Another exciting approach used a machine learning-driven analysis of effectoromes, such as what was done with *C. rodentium*, where multiplexed effector mutants were used to predict the virulence contributions for all individual and combinatorial effectors. Sequential deletion of the top five effectors ranked by predicted virulence impact resulted in complete loss of pathogenicity upon the fifth sequential knockout (Ruano-Gallego et al. 2021). Other innovations that will accelerate the systems-level analysis of effectors include phenotype-guided effector recombination, as was done in *S. Typhimurium* (Chen et al. 2021) and programmable shuttle systems for high-throughput virulence reconstitution in *P. syringae* (Cunnac et al. 2011). These methods enable the identification of the “minimal effectorome,” which is the minimal combination required to maintain the pathogen’s essential pathogenicity.

The *Agrobacterium* transient co-expression system allows rapid identification of ETI-suppressing effectors by detecting reduced ETI responses upon co-infiltration of an ETI-eliciting with candidate ETI-suppressing effectors. For instance, transient expression of *R. solanacearum* effector RipAA in *N. benthamiana* elicits cell death, while co-expression with RipAC, RipI, and RipAU attenuated or abolished HR phenotypes (Yu et al. 2020, Nakano et al. 2021). Immune hyperactivation of regulatory loci offers additional screening avenues. For example, the expression of the autoactive *Arabidopsis* ADR1-L1^D489V^ variant in *N. benthamiana* constitutively triggers HR, which enabled the identification of AvrPtoB as an ETI-suppressor that functions by promoting degradation of ADR1 homologs (Wang et al. 2023). Collectively, these approaches establish robust frameworks for deciphering effector-mediated immune modulation.

Large-scale protein–protein interaction (PPI) mapping represents a powerful tool for systematically investigating NLRome–effectorome interactions. For instance, a high-throughput yeast two-hybrid (Y2H) approach, when integrated with analyses of the *Arabidopsis* self-interactome and the pathogen effector–*Arabidopsis* protein interactome, can reveal interactions between sensor NLRs and their cognate effectors, as well as interactions involving decoy or guardee proteins with sensor NLRs and effectors (Mukhtar et al. 2011, Weßling et al. 2014). For example, AtTCP14 is convergently targeted by multiple effectors and interacts with TIR-NLR-type receptors (AT1G31540). This strategy enables the identification of effectors recognized directly or indirectly by the NLRome.

High-throughput screening for components that trigger the hypersensitive response, offers a powerful systems-level approach to characterize NLRome–effectorome interplay. For example, Taj Arndell et al (Arndell et al. 2024) designed and synthesized an effector library that was co-delivered with a candidate NLR gene into a protoplast population via polyethylene glycol (PEG)-mediated transformation. The resulting protoplast population was then subjected to RNA-seq analysis looking for reduced expression of specific effectors. A lower level of expression would indicate that that effector was recognized by the NLR, causing cell death in the corresponding protoplasts. More recently, Haocheng et al. (Zhu et al. 2025) developed engineered geminivirus replicons that link NLR function to rolling circle replication, enabling high-throughput screening NLR variants capable of re-recognized effectors that initially escaped NLR recognition. In this system, ETI activation suppresses viral replication, providing a convenient readout of NLR function. Together, these systematic platforms represent promising tools for dissecting large-scale NLRome–effectorome networks. Together, these systematic platforms represent promising tools for dissecting large-scale NLRome–effectorome networks.

In recent years, protein language model-based artificial intelligence has demonstrated considerable potential in predicting protein interactions (Zhan et al. 2025). Thus, AI-driven construction of large-scale PPI networks may offer a promising strategy for systematically deciphering NLRome–effectorome interactions.

## Outsmarting effectoromes through the rational design of NLRs

**Figure 3:**
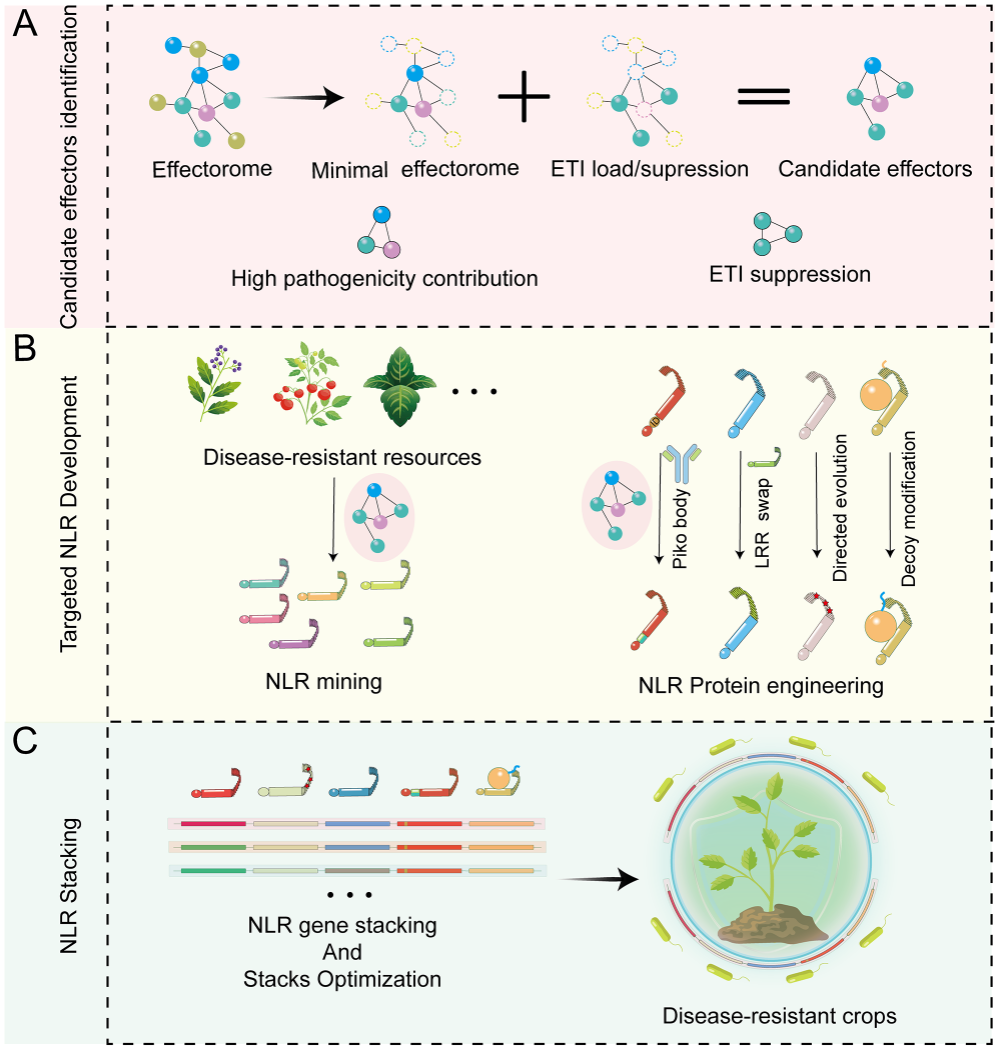
Targeted NLR gene stacking strategy guided by effectorome-NLRome interaction models. The interplay between pathogen effectorome and host NLRome interactions determines ETI load (immunity activation) and ETI-suppression, ultimately shaping crop disease resistance. Strategic NLR stacking amplifies ETI load but requires careful management to avoid compensatory ETI suppression. (A) Effectoromics employs a multifaceted approach to identify effector functional necessity and redundancy, as well as minimal effector repertoires necessary for host-specific virulence. Concurrently, screening for ETI-inducing and ETI-suppressing effectors helps establish effector interaction networks that can be used to identify highly interconnected (i.e. hub) effectors that can be targeted for the development and integration of NLR genes, with the aim of amplifying the ETI load of the target pathogen. (B) The targeted development of NLR resistance genes requires the identification of plants that show hub-effector-associated resistance, and the subsequent mapping and characterization of the corresponding NLR genes. Concurrently, protein engineering techniques can be employed to design and develop specific NLR genes targeting these hub-effectors. (C) The stacking of NLR genes and further optimization should be conducted at the final stage. Ideally, a series of candidate NLR genes would be aggregated into gene clusters to facilitate their application evaluation in various crops, ultimately resulting in crops with efficient, broad-spectrum, and sustainable disease resistance.

The quantitative nature of ETI allows us to model the outcome of a host-pathogen interaction based on the dynamics of ETI elicitation and suppression. This model mechanistically explains two hallmark phenomena in plant-pathogen interactions: the rapid breakdown of monogenic NLR-mediated resistance within a few growing seasons (Brown 2015), and the durable broad-spectrum resistance achieved through strategic stacking of multiple NLR genes (Luo et al. 2021). If we want to use this knowledge to strategically enhance disease resistance, we must amplify ETI load through NLR gene stacking, while counteracting ETI-suppressing effectors, particularly those that remove the selection against highly prevalent effectors with high virulence potential such as the *P. syringae* effector AvrE (Brown 2015, Luo et al. 2021). Therefore, focusing on NLRs that specifically recognize core effectors with high virulence contribution would be reasonable starting point. These NLR genes are excellent candidates for NLR stacking in crops to enhance disease resistance (Fig. 3).

The mining of NLR genes from resistant plant varieties has long constituted a cornerstone of plant immunity research, yielding critical insights into pathogen recognition mechanisms. Positional cloning emerged as a central strategy, leading to the discovery of foundational NLRs including RPS2 and RPM1 (Bent et al. 1994, Grant et al. 1995, Kapos et al. 2019). Reverse genetics complemented this approach by systematically interrogating NLR function through knockout/silencing coupled with infection assays. For instance, a genome-wide NLR silencing screen in *N. benthamiana* coupled with subsequent HR phenotyping upon effector expression, revealed that RRS-Y and Ptr1 are essential for the HR triggered by RipY and RipE1, respectively (Kim et al. 2023a,b).

Building on these methodologies, Genome-wide association study (GWAS) analysis has become a powerful method for identifying disease resistance genes in natural and breeding plant populations (Demirjian et al. 2023). Since its initial application in *A. thaliana* in 2005 (Aranzana et al. 2005), GWAS has been extensively employed to localize resistance loci in plant pathosystems, such as maize (Kump et al. 2011), rice (Zhao et al. 2011), and others. For example, Wu et al. (2025) recently evaluated disease resistance responses across diverse geographical locations and environmental conditions using 1629 wheat germplasm accessions challenged with 12 distinct races of *Puccinia striiformis* f. sp. *Tritici*. Using GWAS, they identified 431 resistance-associated loci, including multiple NLR genes such as *Yr5, Yr6*, and others.

Resistance gene enrichment sequencing (RenSeq) offers yet more possibilities. RenSeq uses oligonucleotide baits derived from known NLR sequences to selectively enrich NLR-family regions from genomic DNA, followed by short- or long-read sequencing to comprehensively characterize the NLRome (Jupe et al. 2013, Witek et al. 2016). By focusing specifically on the NLR gene family, RenSeq achieves greater sequencing depth and facilitates accurate identification of sequence diversity in plants with large genomes and high ploidy levels. This approach effectively identifies NLR polymorphisms in bulked segregant analysis (BSA) populations and chemically mutagenized populations, enabling rapid discovery of novel functional NLR genes (Jupe et al. 2013, Steuernagel et al. 2016). Additionally, NLRome assemblies generated by RenSeq can be integrated with GWAS to accelerate the identification of novel NLR genes from wild germplasm resources (Arora et al. 2019). Lin et al (Van de Weyer et al. 2019, Lin et al. 2023) constructed a *Solanum americanum* pan-NLRome across 52 accessions using RenSeq, correlating NLR diversity with differential recognition of *Phytophthora infestans* RXLR effectors to clone novel NLRs.

Protein engineering strategies have emerged as powerful tools for altering and customizing the recognition functions of NLR proteins, complementing conventional NLR mining approaches (Zdrzałek et al. 2023). Single or a few amino acid substitutions in the recognition domain of an NLR protein can be sufficient to alter its recognition specificity toward effectors. For instance, the rice NLR gene Pik recognizes the *Magnaporthe oryzae* effector AVR-Pik via its integrated HMA domain. Both Pik and Avr-Pik comprise multiple allelic variants, and different Pik alleles exhibit distinct recognition spectra toward Avr-Pik variants (Białas et al. 2018, De la Concepcion et al. 2018). Structure-guided mutations at the binding interface of HMA domain have been shown to alter or broaden the recognition specificity of Pik (Cesari et al. 2022, Maidment et al. 2023, Zhang et al. 2024). More recently, the Geminivirus Replicon-Assisted in Planta Directed Evolution (GRAPE) platform has been established, enabling high-throughput in planta protein evolution (Zhu et al. 2025). Saturation mutagenesis of the HMA domain in Pikm-1, followed by high-throughput screening in planta, identified the S259E-M254D variants of Pikm-1, which confer recognition of all six known Avr-Pik variants. A groundbreaking demonstration of this potential involves nanobody-mediated specificity reprogramming, where replacement of the Pikm1 NLR’s HMA domain with GFP/mCherry-targeting nanobodies generated synthetic “Pikobodies”—engineered receptors conferring resistance against viruses expressing these fluorescent proteins (Kourelis et al. 2023). Complementing this direct recognition engineering, decoy modification strategies exploit indirect effector surveillance mechanisms. The canonical AvrPphB-PBS1-RPS5 detection triad illustrates this paradigm: AvrPphB’s cysteine protease activity cleaves the PBS1 decoy protein to activate RPS5-mediated immunity. Through strategic substitution of PBS1 cleavage site from AvrPphB to AvrRpt2, researchers successfully redirected RPS5 activation to recognize AvrRpt2 effectors (Kim et al. 2016). Wang et al. (2021) developed conditional NLR activation systems using autoactive RRS1-Rslh1 mutants and its suppressor RRS1-RSH/AA. By engineering the RRS1-RSH/AA suppressor protein to undergo effector-dependent degradation, specific pathogen effectors trigger de-repression of RRS1-Rslh1, thereby initiating ETI response. Collectively, these modular engineering platforms, encompassing nanobody fusion, decoy reprogramming, and conditional NLR de-repression, provide unprecidented power and precision for the design of synthetic immune receptors.

## Conclusion

Effectors do not act in isolation. Every pathogenic strain carries a repertoire of effectors that operate as collective assemblages. Many effectors in these repertoires are functionally collaborative or interdependent and share similar molecular functions and/or target the same host cellular compartment or biological process. These features have resulted in an integrated functional effector network that targets the equally sophisticated and robust host immune network. From the species-wide perspective, pangenomic effectoromes may contain thousands of effectors from dozens of distinct families that have the potential to reassort via horizontal gene transfer. The dynamic exchange and birth/death of effectors permit the reassortment of effector repertoires in strains that face extreme selective pressures imposed by the host immune system. Effector assemblages that help strains survive this selective gauntlet are more likely to be maintained and propagated to other strains.

The networked architecture of effectoromes calls for a systems-based framework for interpreting and understanding plant-pathogen interactions. This framework must build on the pioneering gene-for-gene (Flor 1942) and zigzag models (Jones and Dangl 2006) ) and incorporate mechanistic and cellular data integrated with our expanding understanding of pangenome diversity and effectorome-NLRome dynamics. This conceptual evolution should affirm the importance of the collective effector repertoire of each strain (particularly with respect to a strain’s ETI load), the potential confounding impact of effector–effector interactions that will certainly be important for ETI suppression, and the importance of quantitative disease resistance.

The robustness and resilience of effectoromes are crucial for maintaining pathogenic stability, a trait that often enables rapidly evolving pathogens to prevail in long-term interactions with their hosts. From the perspective of plant protection, however, this stability poses a significant challenge for engineering crops with durable and broad-spectrum disease resistance. Systematically studying effectoromes and their interactions with NLRomes offers a promising path forward, particularly when combined with high-throughput NLR mining and novel engineering technologies. This approach allows us to strategically target core nodes within the effector interaction network. By stacking NLRs, we can impose a high ETI load on the pathogen, pushing it beyond a sustainable threshold—one that cannot be easily overcome through natural evolutionary processes—thereby enabling crops to maintain a competitive advantage over pathogens in the long term.
